# Prevalence and Factors Associated With Falls in Individuals Aged 50 Years and Older Living in Urban and Rural Areas in Brazil: A Cross‐Sectional Analysis

**DOI:** 10.1002/pri.70375

**Published:** 2026-11-10

**Authors:** Stéfany Giacomello Piccinin, Merly Fernanda Illera Castellanos, Victória Possebon Sgaria, Bárbara Niegia Garcia de Goulart, Felipe Barreto Schuch, Debora Tornquist, Carine Cristina Callegaro

**Affiliations:** ^1^ Laboratory of Physiology and Rehabilitation Federal University of Santa Maria Santa Maria Brazil; ^2^ Graduate Program in Human Communication Disorders Federal University of Santa Maria Santa Maria Brazil; ^3^ Department of Speech‐Language Pathology Faculty of Health Universidad de Pamplona Pamplona Colombia; ^4^ Faculty of Medicine Graduate Studies Program in Epidemiology Universidade Federal Do Rio Grande Do Sul Porto Alegre Brazil; ^5^ Psychiatry Institute Federal University of Rio de Janeiro Rio de Janeiro Brazil; ^6^ Department of Sports Methods and Techniques Federal University of Santa Maria Santa Maria Brazil; ^7^ Faculty of Health Sciences Universidad Autónoma de Chile Santiago Chile; ^8^ Institute for Physical Activity and Nutrition Deakin University Geelong Australia; ^9^ Institute for Mental and Physical Health and Clinical Translation (IMPACT) Deakin University Geelong Australia; ^10^ Postgraduate Program in Movement Sciences and Rehabilitation Federal University of Santa Maria—UFSM Santa Maria Brazil; ^11^ Department of Physiotherapy and Rehabilitation Graduate Program in Human Communication Disorders Graduate Program in Movement Sciences and Rehabilitation Federal University of Santa Maria Santa Maria Brazil

**Keywords:** elderly, epidemiology, falling accidents, risk factors

## Abstract

**Background and Purpose:**

The factors associated with falls may differ from urban to rural areas. However, there is a paucity of studies investigating this topic. To investigate the prevalence and factors associated with falls in individuals aged 50 years and older living in urban and rural areas.

**Methods:**

This cross‐sectional study analyzed 9402 participants aged 50 and older from the ELSI‐Brasil study baseline in 2015–2016. Of these, 84.7% lived in urban areas. Self‐reported falls in the last 12 months were assessed. The association of falls with age, sex, sleep quality, weakness, weight loss > 3 kg, slow gait, exhaustion, postural balance, osteoporosis, and depression was examined. Weighted prevalence estimates were calculated, and associations with falls were assessed using hierarchical Poisson regression models accounting for the complex sampling design and stratified by area of residence.

**Results:**

The overall prevalence of falls was 22.4% and was higher among urban than rural residents (22.8% vs. 20.1%). Women had a higher prevalence of falls in both urban (PR: 1.64; 95% CI: 1.48–1.82) and rural areas (PR: 1.26; 95% CI: 1.06–1.49). Compared with individuals aged 50–59 years, the prevalence of falls was higher among those aged 70–79 years (urban PR: 1.25; 95% CI: 1.10–1.43; rural PR: 1.54; 95% CI: 1.12–2.12) and aged ≥ 80 years (urban PR: 1.39; 95% CI: 1.17–1.66; rural PR: 1.65; 95% CI: 1.20–2.27). Slow gait (urban PR: 1.41; 95% CI: 1.22–1.63; rural PR: 1.67; 95% CI: 1.12–2.49) and exhaustion (urban PR: 1.46; 95% CI: 1.29–1.65; rural PR: 1.93; 95% CI: 1.51–2.47) were associated with falls in both areas. Among urban residents, poor sleep quality (PR: 1.32; 95% CI: 1.20–1.44), weight loss more than 3 kg (PR: 1.23; 95% CI: 1.07–1.42), postural balance impairment (PR: 1.28; 95% CI: 1.05–1.56), and depression (PR: 1.45; 95% CI: 1.28–1.64) were also associated with a higher prevalence of falls. However, significant interactions with area of residence were observed only for sex and depression.

**Discussion:**

Over 20% of non‐institutionalized Brazilians aged 50 years and older had one or more falls in the past 12 months. Female sex, older age, slow gait speed, and exhaustion were associated with falls in individuals aged 50 years or older in urban and rural areas. Poor sleep quality, weight loss > 3 kg, and depression were associated with falls only in individuals aged 50 years and older in urban areas. The association between female sex and falls was stronger among urban than rural residents, whereas the association between depression and falls differed significantly by area of residence and was observed only among urban residents.

**Trial Registration:**

The ELSI‐Brazil was approved by the Ethics Committee of the Oswaldo Cruz Foundation—Minas Gerais and the process is registered on Plataforma Brasil (CAAE: 34649814.3.0000.5091)

## 1 Introduction

Falls constitute an important public health concern among individuals aged 50 years and older. Globally, falls are responsible for more than 684,000 deaths annually and are associated with substantial short‐ and long‐term disability (World Health Organization 2021). Falls are also associated with fear of falling, fractures, hospitalization, and mortality (Blain et al. 2021). Among community‐dwelling populations, the prevalence of falls varies considerably across settings, with estimates ranging from approximately 16%–30% in rural and urban populations, respectively (Chen et al. 2021). In Brazil, approximately one‐quarter of community‐dwelling older adults report at least one fall annually (Filho et al. 2019). These findings highlight the relevance of investigating the distribution of falls and their associated factors across different population and residential contexts, particularly urban and rural settings.

Falls may arise from the interplay among multiple individual, functional, and health‐related factors. Demographic characteristics, such as female sex and increasing age, have frequently been associated with a greater likelihood of falling (Blain et al. 2021; Filho et al. 2019). With advancing age, functional limitations, including muscle weakness, slow gait, and impaired balance, may further increase susceptibility to falls (Blain et al. 2021; Chen et al. 2021). Reduced muscle strength may impair the ability to respond effectively to postural perturbations, whereas slower gait may reflect diminished physical capacity and has also been associated with recurrent falls (Kyrdalen et al. 2019).

Beyond these functional characteristics, nutritional status and overall physical and psychological health may further contribute to fall susceptibility. Unintentional weight loss has been associated with falls among older adults (Tzeng et al. 2025). Similarly, exhaustion may indicate reduced physical and functional reserves and has been associated with a greater likelihood of falling (Kim et al. 2022). Depression has also been associated with falls and may co‐exist with reduced physical activity and impaired functional performance, potentially further compromising mobility and postural balance (Smith et al. 2025). Osteoporosis is another relevant health‐related factor, given its relationship with falls and the potentially greater clinical consequences of falls among individuals with reduced bone strength (Fong et al. 2023).Sleep‐related factors may represent another relevant dimension of this multifactorial vulnerability. Poor sleep quality has been associated with falls among middle‐aged and older adults (Lee et al. 2021).

The residential context may also be relevant when investigating falls. Studies conducted in different populations have reported differences in the prevalence of falls between rural and urban residents, although findings have not been consistent across settings (Chen et al. 2021; Kim et al. 2020; Zhang et al. 2019). Importantly, differences in fall prevalence between residential contexts may reflect differences in the distribution of individual sociodemographic, health‐related, and functional characteristics. However, relatively few studies have simultaneously examined a broad range of these characteristics when comparing urban and rural populations.

In Brazil, previous analyses of the Brazilian Longitudinal Study of Aging (ELSI‐Brazil) have provided important evidence regarding falls. An analysis of the first wave of ELSI‐Brazil, conducted in 2015–2016, including 4174 adults aged 60 years and older living in urban areas, reported a fall prevalence of 25.1% and identified female sex and older age, among other health‐related characteristics, as factors associated with falls (Filho et al. 2019). More recent ELSI‐Brazil studies have expanded this finding by investigating specific factors among adults aged 50 years and older. For example, an analysis of 8950 participants found an association between poor self‐reported sleep quality and falls among individuals aged 50–84 years (Santos et al. 2024). In another analysis including 8112 adults, low handgrip strength was associated with falls (from their 50s) and at every half‐decade of life, until before the 80 s, even after controlling for confounding variables, including sex, balance, gait speed, and total number of health conditions (Santos et al. 2024), and another analysis including 8374 adults combining the presence of abdominal obesity (waist circumference) and dynapenia (low handgrip strength) was associated with single and recurrent falls (Coelho de Amorim et al. 2024). These studies provide evidence that individual health and functional characteristics are relevant to falls in the Brazilian population.

Nevertheless, previous ELSI‐Brazil studies have generally focused on specific risk factors or populations restricted by age or residential settings. In particular, the previous comprehensive analysis of falls was restricted to adults living in urban areas. Consequently, limited data are available on the prevalence of falls and the simultaneous association with sociodemographic, clinical, and functional characteristics among adults aged 50 years and older living in both urban and rural areas of Brazil.

Therefore, using nationally representative data from ELSI‐Brazil, this study aimed to investigate the prevalence of falls in the previous 12 months and the factors associated with falls among adults aged 50 years and older living in urban and rural areas of Brazil. Specifically, we examined sex, age, weakness, weight loss greater than 3 kg, slow gait, exhaustion, depression, osteoporosis, poor sleep quality, and loss of postural balance as potential factors associated with falls.

## 2 Methods

### 2.1 Study Design

This cross‐sectional study utilized data from the baseline of the Brazilian Longitudinal Study of Aging (ELSI‐Brazil), which interviewed individuals between 2015 and 2016. The study was approved by the Research Ethics Committee of the Oswaldo Cruz Foundation, Minas Gerais (CAAE 34649814.3.0000.5091) and complies with all ethical requirements for conducting scientific studies involving human subjects, including voluntary participation, participant privacy, and confidentiality. Participants who agreed to participate in the study signed an Informed Consent Form.

### 2.2 Participants and Procedures

This study involved people living in 70 municipalities in the five major regions of Brazil, in both rural and urban areas. The study population consisted of individuals aged 50 or older residing in the municipality. The sample size was calculated based on the 2010 census data from the Brazilian Institute of Geography and Statistics and comprised 10,000 people. With this number of participants and assuming a significance level of 95% and a sample design effect of 1.5, it is possible to estimate a prevalence of 1% with a sampling error of 0.25% or a prevalence of 5% with a sampling error of 0.55%. Additionally, with the same significance level and sampling design effect, it is also possible to estimate differences of 2.2% at a prevalence of 10%, with a test power of 80% (Lima‐Costa et al. 2018).

The municipalities were classified into four strata according to their population size, using an optimal stratification criterion proposed previously (Haldane 1945). The criterion was applied to define the size limits of the municipalities and the number of municipalities assigned to each stratum, utilizing the “Stratification” library of the R program. The strata were defined as follows: stratum 1 includes 4420 municipalities with a population of 26,700 or less; stratum 2 includes 951 municipalities with a population between 26,701 and 135,000; stratum 3 includes 171 municipalities with a population between 135,001 and 750,000; and stratum 4 includes 23 municipalities with a population greater than 750,000. The sample was selected in three stages from strata 1, 2, and 3, which include municipalities with up to 750,000 inhabitants. Sample selection for stratum 4, which includes large municipalities, was conducted in two stages.

Data collection involved structured, in‐home individual interviews and physical performance measures were carried out by trained and certified interviewers before the fieldwork began. An adult household member was invited when the participants could not answer the questions. The questionnaires and the operational manual for ELSI‐Brazil are available on the homepage: https://elsi.cpqrr.fiocruz.br/. Participants with missing data for the outcome variable (occurrence of falls) were excluded from the analytical sample of the present study.

### 2.3 Outcomes

The primary outcome was the prevalence of falls in individuals aged 50 years and older in the last year in urban and rural areas, assessed by the following question: “Have you had any falls in the past 12 months?” Those who reported falls were also asked about where the last fall occurred, which could be at home, on the street, or elsewhere (e.g., school, club, sports facility, gym, or elsewhere).

### 2.4 Potential Correlates

Correlated factors associated with falls included sex, age, weakness, weight loss > 3 kg, slow gait, exhaustion, depression, osteoporosis, poor sleep quality, and postural balance impairment. Sex and age were assessed in the home interview. The anthropometric parameters, including weight and height, were obtained in duplicate according to a previous publication (Lima‐Costa et al. 2018). BMI was calculated by the equation: weight (kg)/height^2^ (m^2^) according to WHO (Murray et al. 2020).

Handgrip strength was assessed using a hydraulic hand dynamometer with an adjustable handle (SAEHAN, South Korea) on the dominant upper limb. Participants were asked to apply the most significant force possible in three attempts, with the largest measure used for the analysis. According to a previous study, weakness was defined as handgrip strength in the lowest quintile, adjusted for sex and BMI quartiles (Andrade et al. 2013).

Weight loss was assessed by two questions: In the last 3 months, have you lost weight without dieting? How many kilos have you lost in the last 3 months? A weight loss of more than 3 kg in the previous 3 months was stratified (Andrade et al. 2013).

Gait speed was assessed by using a stopwatch to record the time (in seconds) required to walk 3 m, using the lower of two measurements. Slow gait speed was defined by the highest quintile of time, stratified by sex and height according to a previous study (Andrade et al. 2013), since gait speed can be expected to be reduced in individuals of lesser height (Bohannon 1997) and in women (Samson et al. 2001; Sialino et al. 2021). Slow gait speed was considered for men with a height ≥ 1.65 m who had a gait speed greater than 4.66 seconds, men with a height < 1.65 m who had a gait speed greater than 5.12 seconds, women with a height ≥ 1.53 m who had a gait speed greater than 5.34 seconds, and women with a height < 1.53 m who had a gait speed greater than 5.90 seconds, as well as for those unable to perform the test for both sexes.

Exhaustion was determined through responses to the questions in the Center for Epidemiological Studies Depression Scale (CES‐D) questionnaire: “In the past week, how often did you feel that you could not get going (you started something but could not finish it)?”; “In the past week, how often did your everyday activities require much effort to perform?” Responses of “most of the time” and “always” to at least one of the questions were scored as positive for exhaustion.

Depression was assessed through the question, “Have you ever been diagnosed with depression?” Sleep quality was evaluated using the question, “How do you rate the quality of your sleep?” Answers of “very good” and “good” were considered as good sleep quality, while answers of “fair,” “poor,” and “very poor” were considered as poor sleep quality. Osteoporosis was assessed with the question, “Have you been diagnosed with osteoporosis?.”

Postural balance was evaluated through balance tests according to a previous study (Nakano et al. 2014) based on maintaining three‐foot positions (feet side by side, one foot slightly in front of the other, and one foot behind the other). In this study, postural balance impairment was defined as a score of less than 10 seconds in the feet‐together position. Additionally, participants who did not attempt the task due to perceived risk, incapacitation, or refusal were also considered to have experienced loss of balance.

### 2.5 Statistical Analysis

Descriptive analyses were conducted taking into account the complex sampling design of the ELSI‐Brazil study, incorporating sampling weights, strata, and primary sampling units. Categorical variables were described using weighted relative frequencies (%) and their respective 95% confidence intervals (95% CI), and differences between urban and rural residents were assessed using the Rao–Scott test. For continuous variables, weighted means and their corresponding 95% CI were estimated, and differences between groups were evaluated using simple linear regression models adjusted for the complex sampling design, with area of residence as the independent variable.

The prevalence of falls was described according to the variables of interest using weighted percentages and their respective 95% confidence intervals (95% CI). Differences between urban and rural residents were assessed using the Rao–Scott adjusted Pearson chi‐square test. Associations between potential correlated factors and falls were estimated using Poisson regression models adjusted for the complex sampling design, yielding prevalence ratios (PR) and 95% confidence intervals (95% CI). Analyses were stratified according to the area of residence (urban and rural). In addition, interaction terms between the area of residence and each exposure variable were tested to assess potential effect modification. Adjusted analyses were performed using a hierarchical model (Victora et al. 1997), in which variables from each level were included in the model simultaneously and maintained, with each exposure adjusted for other variables from the same level and the previous level. The hierarchical model was composed of four levels: (1) Sociodemographic factors: gender and age group (Filho et al. 2019); (2) Lifestyle: poor sleep quality (Lee et al. 2021); (3) Functionality factors: weakness, weight loss > 3 kg, slow gait, exhaustion, and postural balance impairment (Blain et al. 2021; Jehu et al. 2022; Zhang et al. 2019); (4) Comorbidities: depression and osteoporosis (Fong et al. 2023; Gambaro et al. 2022). Analyses were performed using Stata software (version 15.0; StataCorp LLC, College Station, TX, USA). A *p*‐value < 0.05 was considered statistically significant.

## 3 Results

This study included 9402 individuals aged 50 years and older, of whom 15.3% lived in rural areas and 84.7% in urban areas. Table 1 presents the characteristics of the study sample stratified by area of residence (urban or rural). Urban residents had higher weighted mean body weight (71.7 kg; 95% CI: 70.8‐72.6 vs. 67.5 kg; 95% CI: 65.1; 70.0) and body mass index (BMI) (28.0 kg/m^2^; 95% CI: 27.8–28.2 vs. 26.8 kg/m^2^; 95% CI: 26.4–27.2) compared with rural residents. In addition, the weighted prevalence of osteoporosis was higher among participants living in urban areas (16.4%; 95% CI: 14.9–18.0) than among those living in rural areas (12.3%; 95% CI: 10.0–15.0). No significant differences were observed for the remaining variables according to area of residence.

**TABLE 1:** Sample description and frequency distribution of individuals living in rural and urban areas.

Variable	Urban area (*N* = 7926) Mean (95% CI)	Rural area (*N* = 1476) Mean (95% CI)	*p*‐value
Age (years)	62.4 (61.5; 63.4)	62.9 (61.9; 63.9)	0.480^a^
Weight (kg)	71.7 (70.8; 72.6)	67.5 (65.1; 70.0)	**0.001** ^a^
Height (m)	1.60 (1.59; 1.61)	1.59 (1.57; 1.60)	0.139^a^
BMI (kg/m^b^)	28.0 (27.8; 28.2)	26.8 (26.4; 27.2)	**< 0.001** ^a^
Handgrip strength (kg)	27.8 (26.9; 28.7)	27.0 (25.9; 28.1)	0.220^a^
Walking test time (s)	4.3 (4.2; 4.5)	4.9 (3.9; 5.8)	0.260^a^
	**% (95% CI)**	**% (95% CI)**	
Sex			
Female	53.9 (50.5; 57.2)	54.3 (50.8; 57.7)	0.855^b^
Male	46.1 (42.8; 49.5)	45.7 (42.3; 49.2)	
Age group			
50–59 years old	48.0 (43.3; 52.6)	45.8 (40.8; 50.8)	0.457^b^
60–69 years old	29.4 (27.4; 31.5)	30.9 (28.2; 33.8)	
70–79 years old	15.4 (13.4; 17.6)	17.1 (14.3; 20.4)	
≥ 80 years old	7.2 (5.9; 8.9)	6.2 (4.9; 7.8)	
Poor quality of sleep			
Yes	45.6 (44.4; 46.8)	44.3 (41.4; 47.3)	0.447^b^
No	54.4 (53.2; 55.6)	55.7 (52.7; 58.6)	
Weakness			
Yes	10.1 (9.0; 11.4)	8.8 (7.1; 10.9)	0.258^b^
No	89.9 (88.6; 91.0)	91.2 (89.1; 92.9)	
Weight loss > 3 kg			
Yes	14.7 (13.7; 15.8)	17.7 (14.5; 21.4)	0.073^b^
No	85.3 (84.3; 86.3)	82.3 (78.6; 85.6)	
Slow gait			
Yes	22.3 (19.7; 25.2)	20.6 (16.2; 25.8)	0.517^b^
No	77.7 (74.9; 80.3)	79.4 (74.2; 83.8)	
Postural balance impairment			
Yes	7.5 (6.1; 9.1)	7.4 (4.1; 13.0)	0.972^b^
No	92.5 (90.9; 93.9)	92.6 (87.0; 95.9)	
Exhaustion			
Yes	27.8 (25.7; 29.9)	31.2 (26.6; 36.2)	0.145^b^
No	72.2 (70.1; 74.3)	68.8 (63.8; 73.4)	
Osteoporosis			
Yes	16.4 (14.9; 18.0)	12.3 (10.0; 15.0)	**0.004** ^b^
No	83.6 (82.1; 85.1)	87.7 (85.0; 90.0)	
Depression			
Yes	18.6 (16.9; 20.4)	18.6 (14.6; 23.3)	0.986^b^
No	81.4 (79.6; 83.1)	81.4 (76.7; 85.4)	
Fall			
Yes	22.8 (21.4; 24.3)	20.1 (18.2; 22.1)	**0.027** ^b^
No	77.2 (75.7; 78.6)	79.9 (77.9; 81.8)	

*Note:* A longitudinal study on the Brazilian Longitudinal Study of Aging (ELSI‐Brasil), 2015–2016 (*n* = 9402). Mean (95% CI): weighted mean (95% confidence interval); % (95% CI): weighted percentage (95% confidence interval). Bold indicates *p* < 0.05 was considered statistically significant.

^a^ Design‐adjusted linear regression.

^b^ Rao–Scott adjusted Pearson chi‐square test.

The overall prevalence of falls in the sample was 22.4%, and it was significantly higher among urban residents (22.8%; 95% CI: 21.4–24.3) compared with rural residents (20.1%; 95% CI: 18.2–22.1) (Table 1). Among participants who reported falls, the distribution of the place of occurrence differed according to area of residence (*p* < 0.001). Urban residents had a higher proportion of falls occurring on the street compared with rural residents (38.0%; 95% CI: 35.0–41.1 vs. 21.8%; 95% CI: 16.3–28.5). In contrast, rural residents showed a higher proportion of falls occurring at home (62.0%; 95% CI: 53.9–69.4 vs. 51.5%; 95% CI: 48.1–55.0) and in other locations (16.2%; 95% CI: 11.2–22.9 vs. 10.5%; 95% CI: 8.5–12.8) than urban residents.

The prevalence of falls was 25% higher among individuals aged 70–79 years living in urban areas (PR = 1.25; 95% CI: 1.10–1.43) and 54% higher among those living in rural areas (PR = 1.54; 95% CI: 1.12–2.12) compared with middle‐aged adults aged 50–59 years. Similarly, individuals aged 80 years and older also had a higher prevalence of falls than middle‐aged adults in both areas of residence (urban: PR = 1.39; 95% CI: 1.17–1.66; rural: PR = 1.65; 95% CI: 1.20–2.27). However, no significant interaction was observed between age group and area of residence (*p* = 0.821). In contrast, sex showed a significant interaction with area of residence (*p* < 0.001). Among urban residents, women had a 64% higher prevalence of falls than men (PR = 1.64; 95% CI: 1.48–1.82), whereas among rural residents, the prevalence of falls was 26% higher in women compared with men (PR = 1.26; 95% CI: 1.06–1.49). Sleep quality did not show a significant interaction with area of residence (*p* = 0.270). However, although the magnitude of the association did not differ significantly between areas, the association between poor sleep quality and falls reached statistical significance only among urban residents, in whom individuals reporting poor sleep quality had a 32% higher prevalence of falls than those without poor sleep quality (PR = 1.32; 95% CI: 1.20–1.44) (Table 2).

**TABLE 2:** Factors associated with fall occurrence in urban and rural locations.

Factors	Urban area				Rural area			
	Fall % (95% CI)	*p*‐value^a^	Adjusted PR (95% CI)	*p*‐value^b^	Fall % (95% CI)	*p*‐value^a^	Adjusted PR (95% CI)	*p*‐value^b^
1st level								
50–59 years old	20.6 (18.5; 23.0)	**< 0.001**	1	**< 0.001**	17.1 (14.8; 19.7)	**0.015**	1	**< 0.001**
60–69 years old	22.4 (20.7; 24.1)		1.06 (0.93; 1.21)		19.8 (16.7; 23.3)		1.19 (0.95; 1.48)	
70–79 years old	26.9 (23.9; 30.0)		**1.25 (1.10; 1.43)**		25.6 (19.1; 33.4)		**1.54 (1.12; 2.12)**	
≥ 80 years old	30.4 (26.6; 34.5)		**1.39 (1.17; 1.66)**		28.1 (20.4; 37.4)		**1.65 (1.20; 2.27)**	
Male	16.8 (15.1; 18.6)	**< 0.001**	1	**< 0.001**	17.9 (16.1; 19.9)	**0.018**	1	**0.011**
Female	28.0 (26.4; 30.0)		**1.64 (1.48; 1.82)**		21.9 (19.0; 25.1)		**1.26 (1.06; 1.49)**	
2nd level								
No poor sleep quality	19.5 (17.8; 21.4)	**< 0.001**	1	**< 0.001**	18.7 (15.2; 22.8)	0.301	1	0.319
Poor sleep quality	26.8 (25.1; 28.6)		**1.32 (1.20; 1.44)**		21.8 (18.8; 25.0)		1.16 (0.87; 1.54)	
3rd level								
No weakness	19.4 (17.9; 20.9)	**< 0.001**	1	0.888	17.7 (15.6; 20.1)	**< 0.001**	1	0.620
Weakness	40.0 (36.8; 43.4)		0.99 (0.84; 1.17)		35.6 (27.3; 44.8)		1.11 (0.74; 1.66)	
No weight loss > 3 kg	21.4 (19.9; 22.9)	**< 0.001**	1	**0.004**	19.4 (17.3; 21.8)	0.561	1	0.148
Weight loss > 3 kg	29.1 (26.6; 31.8)		**1.23 (1.07; 1.42)**		21.1 (16.9; 25.9)		0.77 (0.54; 1.10)	
No slow gait	19.8 (18.4; 21.2)	**< 0.001**	1	**< 0.001**	16.9 (14.9; 19.0)	**< 0.001**	1	**0.012**
Slow gait	31.9 (29.2; 34.6)		**1.41 (1.22; 1.63)**		31.0 (24.5; 38.4)		**1.67 (1.12; 2.49)**	
No postural balance impairment	21.7 (20.3; 23.3)	**< 0.001**	1	**0.015**	19.2 (17.2; 21.3)	**0.001**	1	0.405
Postural balance impairment	36.1 (31.7; 40.7)		**1.28 (1.05; 1.56)**		31.7 (23.9; 40.6)		1.21 (0.77; 1.89)	
No exhaustion	19.1 (17.6; 20.7)	**< 0.001**	1	**< 0.001**	16.2 (13.9; 18.9)	**< 0.001**	1	**< 0.001**
Exhaustion	31.9 (29.5; 34.5)		**1.46 (1.29; 1.65)**		27.4 (23.5; 31.6)		**1.93 (1.51; 2.47)**	
4th level								
No osteoporosis	21.0 (19.6; 22.5)	**< 0.001**	1	0.527	19.3 (17.0; 21.8)	0.141	1	0.596
Osteoporosis	32.1 (29.0; 35.3)		1.04 (0.93; 1.15)		25.0 (18.8; 32.5)		1.14 (0.71; 1.82)	
No depression	19.7 (18.1; 21.3)	**< 0.001**	1	**< 0.001**	19.8 (17.5; 22.3)	0.527	1	0.380
Depression	36.3 (33.4; 39.3)		**1.45 (1.28; 1.64)**		21.7 (17.1; 27.2)		0.85 (0.59; 1.22)	

*Note:* The Brazilian Longitudinal Study of Aging (ELSI‐Brazil), 2015–2016. % (95% CI): weighted percentage (95% confidence interval); PR (95% CI): Prevalence Ratio (95% Confidence Interval). Bold indicates *p* < 0.05 was considered statistically significant.

^a^ Rao–Scott adjusted Pearson chi‐square test.

^b^ Poisson regression adjusted for the complex sampling design.

None of the functionality‐related variables showed a significant interaction with area of residence (weakness: *p* = 0.847; weight loss > 3 kg: *p* = 0.116; slow gait: *p* = 0.353; postural balance impairment: *p* = 0.980; exhaustion: *p* = 0.946). Weakness was not associated with falls among either urban residents (*p* = 0.888) or rural residents (*p* = 0.620). In contrast, both slow gait and exhaustion were associated with a higher prevalence of falls in both areas of residence. Individuals with slow gait had a 41% higher prevalence of falls among urban residents (PR = 1.41; 95% CI: 1.22–1.63) and a 67% higher prevalence among rural residents (PR = 1.67; 95% CI: 1.12–2.49) compared with their counterparts without slow gait. Similarly, individuals reporting exhaustion had a 46% higher prevalence of falls among urban residents (PR = 1.46; 95% CI: 1.29–1.65) and a 93% higher prevalence among rural residents (PR = 1.93; 95% CI: 1.51–2.47) compared with those without exhaustion. Although no significant interaction with area of residence was observed, the associations of weight loss greater than 3 kg and postural balance impairment with falls reached statistical significance only among urban residents. In this group, individuals with weight loss > 3 kg and those with postural balance impairment had a 23% (PR = 1.23; 95% CI: 1.07–1.42) and 28% (PR = 1.28; 95% CI: 1.05–1.56) higher prevalence of falls, respectively, compared with their counterparts without these conditions (Table 2).

Osteoporosis did not show a significant interaction with area of residence (*p* = 0.366) and was not associated with the occurrence of falls among either urban residents (*p* = 0.527) or rural residents (*p* = 0.596). In contrast, a significant interaction was observed between depression and area of residence (*p* = 0.001). The association between depression and falls differed according to area of residence and was observed only among urban residents. In this group, individuals with depression had a 45% higher prevalence of falls than those without depression (PR = 1.45; 95% CI: 1.28–1.64) (Table 2).

## 4 Discussion

Urban residents showed a greater prevalence of falls than rural residents. Among urban adults, depression, poor quality of sleep, weight loss more than 3 kg, and postural balance impairment were associated with a higher prevalence of falls. However, interaction analyses indicated that only the association between depression and falls differed significantly according to area of residence, whereas the associations involving poor sleep quality, weight loss more than 3 kg, and postural balance impairment did not. This study also identified that age, female sex, exhaustion, and slow gait are associated with falls for urban and rural residents, but the association between female sex and falls is stronger among urban than rural residents. The greater prevalence of street‐related falls among urban residents may be related to greater exposure to pedestrian environments during daily activities and transportation, since (Pimentel et al. 2018), using the data from ELSI‐Brazil found an association between falls and characteristics of the urban environment, particularly defective sidewalks and fear of crossing streets. In contrast, the higher occurrence of falls in other outdoor locations among rural residents may reflect greater exposure to outdoor environments during daily activities and work. This interpretation is supported by a study of rural older adults in which falls frequently occurred on roads (20.6%) and at workplaces, particularly farmland (20.1%), with walking and working being the most common activities at the time of the fall (Zhang et al. 2022).Thus, the observed differences in fall location may reflect differences in environmental exposure and daily activities between urban and rural settings.

The association between depression and falls among individuals aged 50 years and older living in urban areas is consistent with previous findings from ELSI‐Brazil, where depression was independently associated with falls in this same age group but limited to those living in urban areas (PR = 1.53, 95% CI 1.32–1.77) (Pimentel et al. 2018). In the present study, a significant interaction between depression and area of residence was observed, supporting a difference in the association according to residential setting. The association was observed among urban residents but not among rural residents. A previous ELSI‐Brazil analysis including 8950 adults aged 50 years and older from both urban and rural areas, analyzed together, found that poor self‐reported sleep quality was associated with falls among individuals up to 84 years of age (Santos et al. 2024). To our knowledge, the present study is the first to examine this association separately according to urban and rural residence. We found that poor sleep quality was associated with falls among urban residents, whereas the association did not reach statistical significance among rural residents. However, the interaction between sleep quality and area of residence was not statistically significant, indicating that the observed pattern should not be interpreted as evidence of a difference in the association between urban and rural residents. One possible explanation for this context‐specific association is that sleep problems may be influenced by characteristics of the urban environment. An ELSI‐Brazil study of 5719 adults aged ≥ 60 years found that neighborhood problems, including poor street conditions and traffic noise, were associated with greater odds of sleep problems, particularly poor sleep quality (Sutil et al. 2024). These findings suggest that characteristics of urban neighborhoods may contribute to poorer sleep; however, given the absence of a statistically significant interaction, this potential explanation for the observed pattern should be interpreted cautiously.

In the present study, weight loss > 3 kg was also associated with falls only among urban residents. However, the interaction between weight loss more than 3 kg and area of residence was not statistically significant; therefore, these findings do not provide evidence that the association differed between urban and rural residents. Unintentional weight loss may reflect inadequate nutritional status, loss of muscle mass, or declining overall health, which may increase susceptibility to falls (Tzeng et al. 2025).

In this study, impaired postural balance was associated with falls among urban residents aged 50 years and older, whereas the association did not reach statistical significance among rural residents. However, the interaction between postural balance impairment and area of residence was not statistically significant. Therefore, the observed pattern should not be interpreted as evidence of a difference in the association between urban and rural residents. Our findings should therefore be interpreted as evidence of an association observed in the urban stratum rather than as evidence of an urban‐specific effect. Among older adults in China, decreased balance was associated with falls in both urban (OR = 3.57) and rural areas of Shantou (OR = 4.25) (Chen et al. 2021). Importantly, our study did not investigate the specific causes or mechanisms of falls; rather, postural balance was assessed using a physical performance test. Therefore, impaired postural balance should be interpreted as an associated factor rather than a demonstrated cause of falls.

As expected, based on previous studies (Blain et al. 2021; Filho et al. 2019), older age and female sex were associated with falls in both urban and rural residents in our study. However, the association between female sex and falls was stronger among urban than rural residents. In addition, exhaustion was associated with falls among individuals aged 50 years and older in both urban and rural settings, consistent with findings from a study of 447 community‐dwelling older adults, in which exhaustion was the only component of the Fried frailty phenotype significantly associated with a history of falls (Umegaki et al. 2019). Further support comes from a 12‐year longitudinal study of 3122 older adults, which found that exhaustion was independently associated with subsequent falls (OR = 1.28, 95% CI 1.10–1.51) (Kim et al. 2022).

In the present study, slow gait was associated with having experienced at least one fall in the previous year among both urban and rural individuals aged 50 years and older. This finding is consistent with a previous umbrella review reporting moderate evidence that gait speed may be useful for predicting falls in older adults (Beck Jepsen et al. 2022), although our findings demonstrate an association rather than a predictive or causal relationship.

This study has important strengths and limitations. One strength is the use of a population‐based sample from the Brazilian Longitudinal Study of Aging (ELSI‐Brazil), a household survey conducted with a nationally representative sample of people aged 50 years and older. However, the cross‐sectional design of the present analysis precludes establishing temporal or causal relationships between the identified factors and falls (Savitz and Wellenius 2023). Additionally, the smaller proportion of participants living in rural areas may have limited the statistical power of analyses involving rural residents. Further studies should also consider other factors potentially associated with falls that were not assessed in the present study, such as fractures, vision impairment, hearing impairment (Ou et al. 2026), occupation (Zhang et al. 2022), and medication use, particularly antidepressants, sedatives, anxiolytics, and diuretics (Rezende et al. 2012).

## 5 Implications for Physiotherapy Practice

The study findings suggest that older age, female sex, exhaustion, and slow gait speed were associated with falls in both rural and urban areas, with a stronger association between female sex and falls among urban than rural residents. Among urban residents, depression, poor sleep quality, weight loss > 3 kg, and impaired postural balance were also associated with falls.

These factors may help identify individuals who could benefit from fall screening and further assessment. The findings also suggest that residential context may be relevant when considering fall‐related factors, particularly given the significant interaction observed for female sex and depression. However, differences in associations should not be inferred for factors for which the interaction with area of residence was not statistically significant.

## Funding

The ELSI‐Brazil was supported by the Brazilian Ministry of Health: DECIT/SCTIE (Grants: 404965/2012‐1 and TED 28/2017); COPID/DECIV/SAPS (Grants: 20836, 22566, 23700, 25560, 25552, and 27510).

## Ethics Statement

The ELSI‐Brazil was approved by the Ethics Committee of the Oswaldo Cruz Foundation—Minas Gerais and the process is registered on Plataforma Brasil (CAAE: 34649814.3.0000.5091).

## Consent

Participants signed separate informed consent forms for each of the research procedures and authorized access to the corresponding secondary databases.

## Conflicts of Interest

The authors declare no conflicts of interest.

## Permission to Reproduce Material From Other Sources

Access to ELSI‐Brazil data Access to ELSI‐Brazil data is granted through https://elsi.cpqrr.fiocruz.br/en/home‐english/en‐data‐access/.

## Data Availability

The data that support the findings of this study are openly available in the Brazilian Longitudinal Study of Aging (ELSI‐Brazil) at https://elsi.cpqrr.fiocruz.br/en/home‐english/.
